# Macroscopically Visible Trichina

**Published:** 1898-05

**Authors:** 


MACROSCOPICALLY VISIBLE TRICHINA.—According to the *Deutsche Theirärztliche Wochenschrift*, Dr. Simon, in Gorlitz, noticed a three-year-old sow, over the muscles of whose body, especially on the diaphragm, ribs, and muscles of the tongue, innumerable calcinous spots of from ½ to 1 mm. in size were plainly visible to the naked eye. The muscles of the heart were entirely free. The microscopic investigation, when acetic acid had been applied, showed long, spindle-shaped calcinations lying within the muscular tissue, with complete disappearance of the contractile substance of the latter. In some of the preparations eye-shaped capsules were plainly visible.—*Idem.*

